# Supplementary material for: Rapid and green detection of manganese in electrolytes by ultraviolet-visible spectrometry to control pollutant discharge
Source: PLoS One. 2022 Feb 25;17(2):e0264532. doi: 10.1371/journal.pone.0264532 (PMC8880954; doi:10.1371/journal.pone.0264532)
Supplement: S1 File — (DOCX) [file pone.0264532.s001.docx]

Electronic Supplementary Material

Rapid and green detection of manganese in electrolytes by ultraviolet-visible spectrometry to control pollutant discharge

Zhehua Xue ^1,2^; Lei Li ^1,2⁎^

^1^State Key Laboratory of Pollution Control and Resource Reuse, Tongji University, Shanghai, China

^2^Shanghai Institute of Pollution Control and Ecological Security, Shanghai, China

⁎ Corresponding author

E-mail address: lileitongji@126.com

TABLE OF CONTENTS

Page 2 Supplementary Fig S1

Page 2 Supplementary Fig S2

Page 3 Supplementary Fig S3

Page 3 Supplementary Fig S4

Page 4 Supplementary Fig S5

Page 4 Supplementary Fig S6

Page 5 Supplementary Fig S7

Page 6 Supplementary Table S1

Page 6 Supplementary Table S2

Page 7 Supplementary Table S3


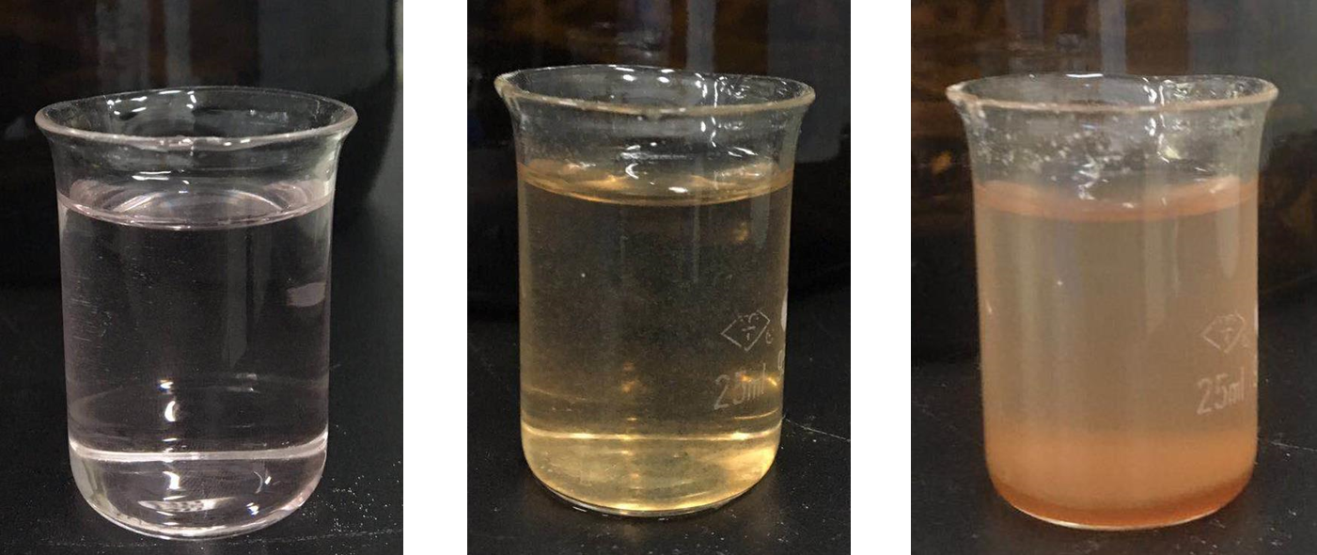


**(a)**

**(b)**

**(c)**

**Fig S1.** **Photographs of a MnSO_4_ solution (80.0 g/L) stored for different times.** (a) newly prepared, (b) stored in open air for one day and (c) stored in open air for one week (ambient temperature, without pH adjustment).





**Fig S2.** **Spectra of 40.0 g/L (Mn^2+^) MnSO_4_ solution at different pH values.**





**Fig S3.** **Size distribution of particles in MnSO_4_ solution.** (a) pH=1.8 and (b) pH=7.4.


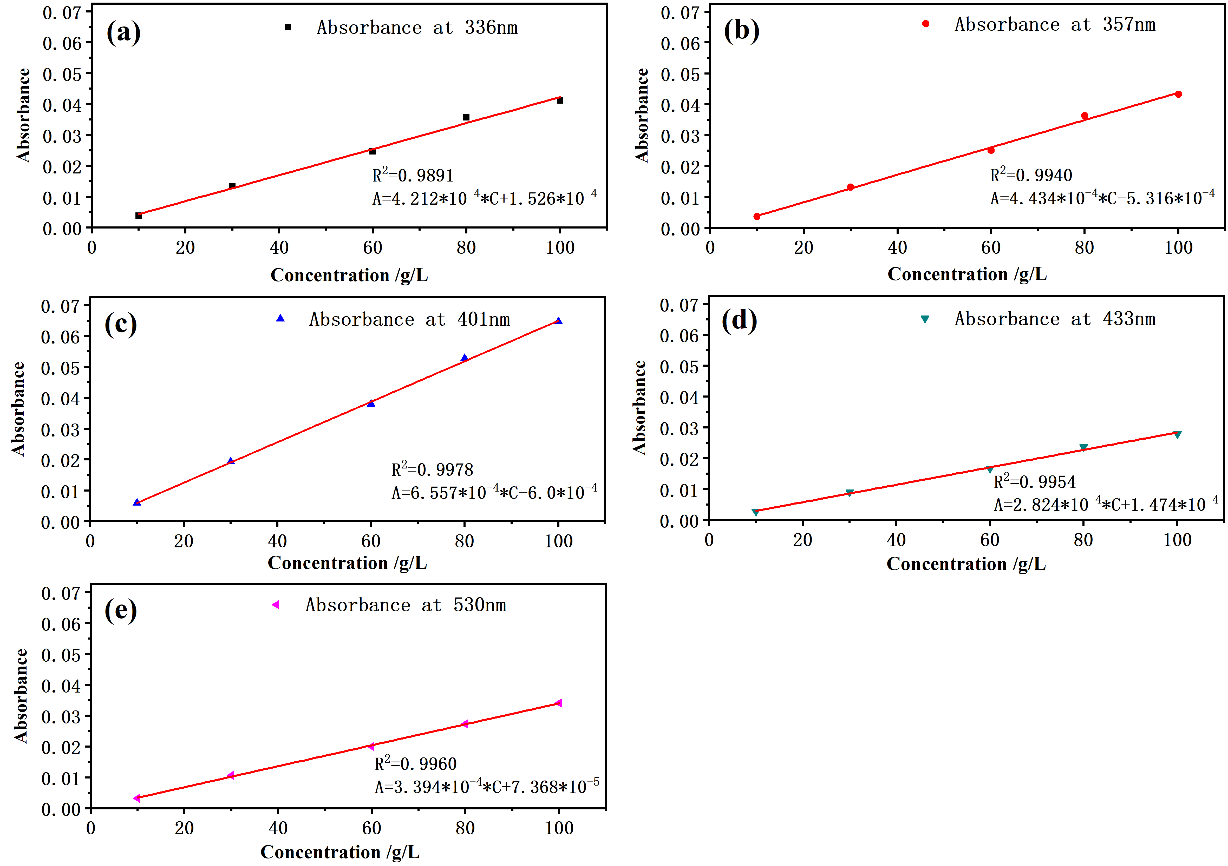


**Fig S4.** **Fitted absorbance versus concentration curves at different wavelengths.** With the correlation coefficient and fitting formula (regression coefficient in the formula represents absorption coefficient).





**Fig S5.** **Spectra of MnSO_4_ solutions.** With the same concentration but different pH values after being stored for 30.0 min in open air.

**Fig S6.** **Spectra of MnSO_4_ solutions and concentrations of Mn^2+^ calculated with revised absorbance.** (a) 16.0, 17.0, and 18.0 g/L and (b) 38.0, 39.0, and 40.0 g/L (absorption coefficient is obtained by the fitting result in Fig S4 at 401 nm).





**Fig S7.** **Absorption spectra of MnSO_4_ solution with and without coexisting substances.**

**Table S1.** **Absorbance in cuvettes of different lengths (each measured 20 times) and standard deviation.**

| OPL/cm | Absorbance | | | | | | | | | | Standard deviation | |
| --- | --- | --- | --- | --- | --- | --- | --- | --- | --- | --- | --- | --- |
| 1 | 0.0317 | 0.0318 | 0.0318 | 0.0321 | 0.0318 | 0.0318 | 0.0319 | 0.0319 | 0.032 | 0.032 | | 0.00014 |
|  | 0.032 | 0.032 | 0.032 | 0.0318 | 0.0317 | 0.0317 | 0.0317 | 0.0317 | 0.0317 | 0.0317 | |  |
|  |  |  |  |  |  |  |  |  |  |  | |  |
| 2 | 0.0614 | 0.0618 | 0.0614 | 0.0615 | 0.0614 | 0.0614 | 0.0614 | 0.0614 | 0.0614 | 0.0614 | | 0.00012 |
|  | 0.0613 | 0.0614 | 0.0613 | 0.0612 | 0.0613 | 0.0614 | 0.0614 | 0.0614 | 0.0614 | 0.0616 | |  |
|  |  |  |  |  |  |  |  |  |  |  | |  |
| 3 | 0.0914 | 0.0913 | 0.0914 | 0.0913 | 0.0913 | 0.0913 | 0.0914 | 0.0914 | 0.0914 | 0.0915 | | 0.00014 |
|  | 0.0915 | 0.0914 | 0.0915 | 0.0914 | 0.0916 | 0.0916 | 0.0917 | 0.0917 | 0.0917 | 0.0916 | |  |
|  |  |  |  |  |  |  |  |  |  |  | |  |
| 5 | 0.157 | 0.157 | 0.157 | 0.1569 | 0.157 | 0.1569 | 0.157 | 0.1569 | 0.1569 | 0.1568 | | 0.00013 |
|  | 0.1567 | 0.1567 | 0.1567 | 0.1567 | 0.1567 | 0.1567 | 0.1567 | 0.1567 | 0.1568 | 0.1568 | |  |
|  |  |  |  |  |  |  |  |  |  |  | |  |
| 10 | 0.3057 | 0.3055 | 0.3057 | 0.3056 | 0.3057 | 0.3057 | 0.3058 | 0.306 | 0.306 | 0.306 | | 0.00014 |
|  | 0.306 | 0.3057 | 0.3058 | 0.3057 | 0.3058 | 0.3057 | 0.3057 | 0.3057 | 0.3057 | 0.3057 | |  |

**Table S2.** **Concentrations of coexisting substances in qualified electrolyte sampled from industry.**

| Substance | Fe | Zn | Ni | Co | As | Cu |
| --- | --- | --- | --- | --- | --- | --- |
| Concentration | 0.2 mg/L | 3.0 mg/L | 0.5 mg/L | 0.5 mg/L | 0.6 mg/L | 0.5 mg/L |
| Substance | Si | P | Mg | Ca | Se | (NH_4_)_2_SO_4_ |
| Concentration | 0.01 mg/L | 0.2 mg/L | 15.0 g/L | 2.0 g/L | 60.0 mg/L | 120.0 g/L |

**Table S3.** **Concentrations calculated with the revised and unrevised absorbance compared with the prepared concentrations.**

| Prepared concentration | Unrevised absorbance (401nm) | Calculated concentration (unrevised) | Relative error (unrevised) | Absorbance (386nm) | Absorbance (411nm) | Revised absorbance (401nm) | Calculated concentration (revised) | Relative error (revised) |
| --- | --- | --- | --- | --- | --- | --- | --- | --- |
| 40.0 g/L | 0.322 | 49.2 | 23.0 % | 0.084 | 0.098 | 0.230 | 40.7 | 1.7 % |
| 36.0 g/L | 0.300 | 45.8 | 27.2 % | 0.087 | 0.098 | 0.206 | 36.5 | 1.3 % |
| 20.0 g/L | 0.213 | 32.6 | 63.0 % | 0.101 | 0.101 | 0.112 | 20.2 | 1.0 % |
| 16.0 g/L | 0.174 | 26.6 | 66.3 % | 0.085 | 0.083 | 0.088 | 16.1 | 0.6 % |
